# Federated Learning for Decentralized Artificial Intelligence in Melanoma Diagnostics

**DOI:** 10.1001/jamadermatol.2023.5550

**Published:** 2024-02-07

**Authors:** Sarah Haggenmüller, Max Schmitt, Eva Krieghoff-Henning, Achim Hekler, Roman C. Maron, Christoph Wies, Jochen S. Utikal, Friedegund Meier, Sarah Hobelsberger, Frank F. Gellrich, Mildred Sergon, Axel Hauschild, Lars E. French, Lucie Heinzerling, Justin G. Schlager, Kamran Ghoreschi, Max Schlaak, Franz J. Hilke, Gabriela Poch, Sören Korsing, Carola Berking, Markus V. Heppt, Michael Erdmann, Sebastian Haferkamp, Konstantin Drexler, Dirk Schadendorf, Wiebke Sondermann, Matthias Goebeler, Bastian Schilling, Jakob N. Kather, Stefan Fröhling, Titus J. Brinker

**Affiliations:** 1 Digital Biomarkers for Oncology Group, National Center for Tumor Diseases (NCT), German Cancer Research Center (DKFZ), Heidelberg, Germany; 2 Department of Dermatology, Venereology and Allergology, University Medical Center Mannheim, Ruprecht-Karls University of Heidelberg, Mannheim, Germany; 3 Skin Cancer Unit, German Cancer Research Center (DKFZ), Heidelberg, Germany; 4 DKFZ Hector Cancer Institute at the University Medical Center Mannheim, Mannheim, Germany; 5 Skin Cancer Center at the University Cancer Center and National Center for Tumor Diseases Dresden, Department of Dermatology, University Hospital Carl Gustav Carus, Technische Universität Dresden, Dresden, Germany; 6 Department of Dermatology, University Hospital (UKSH), Kiel, Germany; 7 Department of Dermatology and Allergy, University Hospital, LMU Munich, Munich, Germany; 8 Dr Phillip Frost Department of Dermatology and Cutaneous Surgery, Miller School of Medicine, University of Miami, Miami, Florida; 9 Department of Dermatology, University Hospital Erlangen, Comprehensive Cancer Center Erlangen–European Metropolitan Region Nürnberg, CCC Alliance WERA, Erlangen, Germany; 10 Department of Dermatology, Venereology and Allergology, Charité–Universitätsmedizin Berlin, Corporate member of Freie Universität Berlin and Humboldt-Universität zu Berlin, Berlin, Germany; 11 Department of Dermatology, University Hospital Regensburg, Regensburg, Germany; 12 Department of Dermatology, Venereology and Allergology, University Hospital Essen, Essen, Germany; 13 Department of Dermatology, Venereology and Allergology, University Hospital Würzburg and National Center for Tumor Diseases (NCT) WERA, Würzburg, Germany; 14 Else Kroener Fresenius Center for Digital Health, Technical University Dresden, Dresden, Germany; 15 Department of Translational Medical Oncology, National Center for Tumor Diseases (NCT) Heidelberg and German Cancer Research Center (DKFZ), Heidelberg, Germany

## Abstract

**Importance:**

The development of artificial intelligence (AI)–based melanoma classifiers typically calls for large, centralized datasets, requiring hospitals to give away their patient data, which raises serious privacy concerns. To address this concern, decentralized federated learning has been proposed, where classifier development is distributed across hospitals.

**Objective:**

To investigate whether a more privacy-preserving federated learning approach can achieve comparable diagnostic performance to a classical centralized (ie, single-model) and ensemble learning approach for AI-based melanoma diagnostics.

**Design, Setting, and Participants:**

This multicentric, single-arm diagnostic study developed a federated model for melanoma-nevus classification using histopathological whole-slide images prospectively acquired at 6 German university hospitals between April 2021 and February 2023 and benchmarked it using both a holdout and an external test dataset. Data analysis was performed from February to April 2023.

**Exposures:**

All whole-slide images were retrospectively analyzed by an AI-based classifier without influencing routine clinical care.

**Main Outcomes and Measures:**

The area under the receiver operating characteristic curve (AUROC) served as the primary end point for evaluating the diagnostic performance. Secondary end points included balanced accuracy, sensitivity, and specificity.

**Results:**

The study included 1025 whole-slide images of clinically melanoma-suspicious skin lesions from 923 patients, consisting of 388 histopathologically confirmed invasive melanomas and 637 nevi. The median (range) age at diagnosis was 58 (18-95) years for the training set, 57 (18-93) years for the holdout test dataset, and 61 (18-95) years for the external test dataset; the median (range) Breslow thickness was 0.70 (0.10-34.00) mm, 0.70 (0.20-14.40) mm, and 0.80 (0.30-20.00) mm, respectively. The federated approach (0.8579; 95% CI, 0.7693-0.9299) performed significantly worse than the classical centralized approach (0.9024; 95% CI, 0.8379-0.9565) in terms of AUROC on a holdout test dataset (pairwise Wilcoxon signed-rank, *P* < .001) but performed significantly better (0.9126; 95% CI, 0.8810-0.9412) than the classical centralized approach (0.9045; 95% CI, 0.8701-0.9331) on an external test dataset (pairwise Wilcoxon signed-rank, *P* < .001). Notably, the federated approach performed significantly worse than the ensemble approach on both the holdout (0.8867; 95% CI, 0.8103-0.9481) and external test dataset (0.9227; 95% CI, 0.8941-0.9479).

**Conclusions and Relevance:**

The findings of this diagnostic study suggest that federated learning is a viable approach for the binary classification of invasive melanomas and nevi on a clinically representative distributed dataset. Federated learning can improve privacy protection in AI-based melanoma diagnostics while simultaneously promoting collaboration across institutions and countries. Moreover, it may have the potential to be extended to other image classification tasks in digital cancer histopathology and beyond.

## Key Points

**Question:**

Can a privacy-preserving federated learning approach achieve comparable diagnostic performance to classical centralized learning approaches for artificial intelligence–based melanoma diagnostics?

**Findings:**

In a consecutive multicenter diagnostic study involving 1025 whole-slide images of clinically melanoma-suspicious skin lesions from 923 patients, a melanoma-nevus classifier developed using classical centralized learning significantly outperformed the federated model in terms of area under the receiver operating characteristic curve on a holdout test dataset but performed significantly worse than the federated model on an external test dataset.

**Meaning:**

Federated learning has the potential to achieve at least on-par performance to classical centralized learning approaches while simultaneously promoting collaboration across institutions and countries.

## Teaser

This diagnostic study investigates the performance of a privacy-preserving federated learning approach vs a classical centralized and ensemble learning approach for artificial intelligence–based melanoma diagnostics.

## Introduction

Convolutional neural networks—deep neural networks most commonly applied to image classification—have shown promise in improving diagnostic accuracy for various diseases,^1,2,3^ including melanoma.^4,5,6,7^ Melanoma is the leading cause of skin cancer deaths worldwide.^8^ Early-stage detection increases the survival chances of affected patients significantly but is challenging due to frequent morphological overlap between melanoma and atypical nevi.^9,10^ In experimental settings, convolutional neural networks have achieved performance on par or even superior to that of human experts for both dermatological^11,12,13,14^ and histopathological^15,16^ classification tasks. These results suggest that artificial intelligence (AI) has the potential to revolutionize the diagnosis of melanoma in offering more accurate detection.

Nonetheless, AI models are highly data dependent, meaning that their performance correlates with the size and diverseness of the training set. The more diverse data an AI model is trained on, the more likely it is to perform well.^17,18,19^ Therefore, to develop AI algorithms, patient data are typically transferred to one site for training and testing and stored in a centralized way (known as classical centralized learning). However, in the medical field, ensuring patient data confidentiality is of utmost importance; consequently, sharing patient data is heavily regulated. Thus, the transfer of patient data to an external facility to generate the envisaged algorithms can raise serious privacy concerns. Alternatively, institutions can use their own data and computing power to develop separate AI algorithms, whose decisions are subsequently merged into one (known as ensemble learning). However, clinical settings often face computational resource constraints, making it challenging to run complex ensemble models in real time. These framework conditions pose difficulties for collaboration and data collection, particularly in multicenter studies or international research collaborations.

To address these challenges, new approaches, such as federated learning (FL),^20,21^ have been developed to enable the decentralized training of AI algorithms using data kept at their origin, while requiring less computational power on site. FL involves each institution training its own model with its own data, while communication and aggregation are executed by a central coordinator.

Previous studies have examined the use of FL in diagnosing melanoma^22,23^ and other medical applications.^24,25,26,27^ While Bdair et al^22^ and Agbley et al^23^ have demonstrated the promise of FL for classifying retrospective melanoma data, to our knowledge no study has evaluated FL leveraging prospectively collected, clinically representative distributed melanoma data nor externally validated the performance of the proposed classifiers. These gaps in the existing literature highlight the need for further research to explore the effectiveness of FL for melanoma diagnostics when leveraging prospective data and to assess the generalizability of the respective classifiers. Therefore, we developed a model using a decentralized FL approach for the binary classification of invasive melanomas (IMs) and nevi based on histopathological whole-slide images (WSIs) and directly compared it retrospectively with the classical centralized and ensemble learning on both a holdout and an external test dataset using prospectively collected, clinically representative distributed data from 6 German university hospitals.

## Methods

### Ethics Statement and Reporting Standards

Ethics approval was obtained from the ethics committee at the Technical University of Dresden, the Friedrich-Alexander University Erlangen-Nuremberg, the LMU Munich, the University of Regensburg, and the University Hospital Wuerzburg. Patients provided written informed consent. This work was performed in accordance with the Declaration of Helsinki. The Standards for Reporting of Diagnostic Accuracy (STARD) reporting guidelines were followed for the reporting of this study (eTable 2 in Supplement 1).^28^

### Patient Cohorts and Slide Acquisition

Hematoxylin-eosin–stained reference slides of skin lesions were prospectively acquired at 6 German university hospitals (Berlin, Dresden, Erlangen, Munich, Regensburg, Wuerzburg) between April 2021 and February 2023. Study participants had to be at least 18 years old and were required to have clinically melanoma-suspicious skin lesions. Lesions were not allowed to have been previously biopsied or located under the fingernails or toenails. Diagnostic labels were histopathologically confirmed by at least 1 reference dermatopathologist at the corresponding hospital as part of routine clinical practice. In collision cases involving multiple tumors, the label of the larger tumor region was assigned. Only histopathologically confirmed IMs and nevi were eligible for this study.

### WSI Preprocessing

An Aperio AT2 DX slide scanner (Leica Biosystems) was used to digitize the hematoxylin-eosin−stained reference slides of all enrolled patients at ×40 magnification, producing WSIs with a resolution of 0.25 μm/pixel to generate patches for training and testing. After manually annotating the area of the epidermis (M. Schmitt), the region of interest was tessellated into downscaled square patches. Each patch had a uniform edge length of 224 pixels, corresponding to 103.04 μm. WSI annotation and tessellation were performed using QuPath, version 0.2.3.^29^ Additionally, blur detection was implemented with custom code written in Python, version 3.7.0 (Python Software Foundation). A patch was classified as blurry if it had a Laplacian below a manually set threshold of 510 and subsequently discarded.

### Model Development

ResNet18 pretrained on ImageNet was used to train one model with FL, one with centralized learning, and one with ensemble learning. A small architecture was used to limit training and inference time and streamline the experimental procedures. The tree-structured Parzen estimator^30^ was used to choose the hyperparameters to maximize the area under the receiver operating characteristic curve (AUROC) at lesion level for a validation set. For each approach, the learning rate, number of training epochs, amount of data used in 1 epoch per WSI, and, for FL specifically, the frequency of weight exchange were tuned for an equal number of optimization steps using the Python library Optuna.^31^ During this process, 30% of the training data served as the validation set, and the training followed Leslie Smith’s 1-cycle policy, which involves training the model with a gradually increasing learning rate for the first half of the training cycle, followed by a gradual decrease in the learning rate for the second half.^32^ During inference, the confidence value of every patch of a WSI was interpreted as the probability for classification as IM or nevus. The average of these probabilities was the final probability for each WSI.

For the federated approach, data from hospitals 1 to 5 were leveraged. Each hospital’s model was trained for a certain time interval with the same hyperparameters. The time interval was based on a synchronization factor, which was tuned during training and was proportional to the size of the dataset of the respective hospital. After each interval, model weights were collected and merged into a new model using a weighted average. The assigned weights were proportional to the amount of data available during training. Subsequently, the new model was (re)distributed to every hospital to continue training. Since communication between the participants in this approach was not the focus, this process was only simulated on 1 computational unit.

For the centralized approaches, the model Hfull represents the model that was trained using data from hospitals 1 to 5. The remaining 5 models (models H1, H2, H3, H4, and H5) were trained by excluding the data of hospitals 1, 2, 3, 4, or 5, respectively.

For the ensemble approach, 5 classifiers were trained separately using only 1 of the 5 training sets from hospitals 1 to 5 with individual hyperparameters. For inference, each model computed a probability for a given input. All 5 probabilities were subsequently averaged to calculate the final prediction. Training and inference were implemented in Python, version 3.7.0, using PyTorch, version 1.13.0,^33^ and fastai, version 2.7.10.^34^

### Statistical Analysis

Two-sided χ^2^ tests were used to identify significant differences between the training and test datasets. The AUROC served as the primary end point for evaluating the performance of the developed models. Secondary end points included balanced accuracy, sensitivity, and specificity. The mean values of the corresponding metrics were calculated using 1000 iterations of bootstrapping to reduce the impact of stochastic events. The 95% CIs were calculated using the nonparametric percentile method.^35^ For statistical comparisons of the AUROCs, pairwise 2-sided Wilcoxon signed-rank tests were applied. A significance level of *P* < .05 was set for all analyses. Significance levels were adjusted to 0.025 (*m* = 2) or 0.01 (*m* = 5) according to Bonferroni correction in case of multiple tests. Statistical analysis was performed in SPSS, version 29.0.0.0 (IBM Corporation).

## Results

### Number of Eligible Slides and Patients

**Figure 1.**
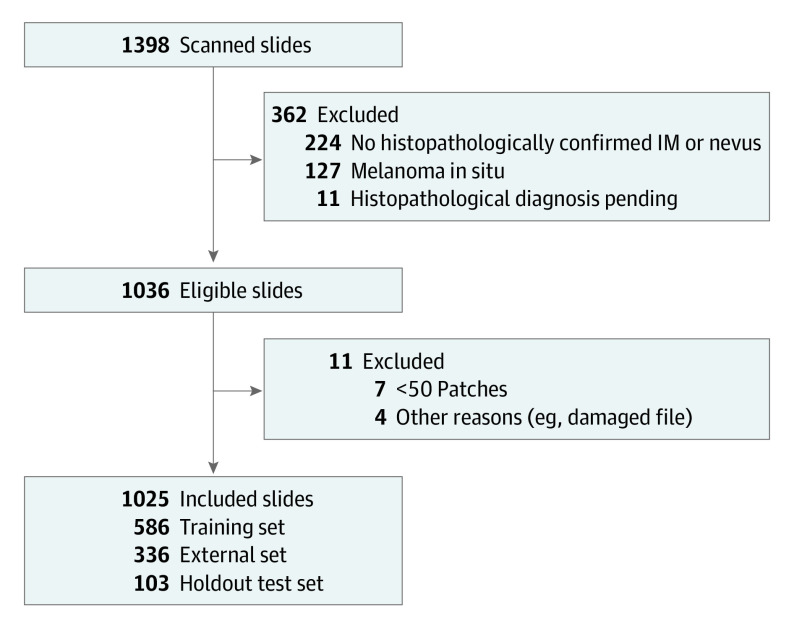
Flowchart of the Slide Inclusion Process Slides were excluded from the analysis if there was no histopathologically confirmed label available or if the lesion proved to be neither invasive melanoma (IM) nor nevus (in situ tumors or other diagnoses, eg, basal cell carcinoma, squamous cell carcinoma). In addition, slides that exhibited fewer than 50 epidermal patches or other technical issues were removed.

A total of 1025 slides from 923 patients, consisting of 388 IMs and 637 nevi, were included in the analysis (Table 1). A further 373 slides were excluded for not meeting the predefined inclusion criteria of this study (eg, in situ tumors; Figure 1). A total of 548 755 patches were derived from the eligible slides (296 141 IMs, 252 614 nevi) for training and testing purposes (eTable 1 in Supplement 1).

**Table 1.** Characteristics of the Study Sample

Hospital	No.		
	Slides (patients)	Invasive melanomas	Nevi
1	71 (62)	19	52
2	97 (86)	56	41
3	107 (103)	59	48
4	178 (157)	37	141
5	236 (215)	75	161
6	336 (300)	142	194
Total	1025 (923)	388	637

### Patient Characteristics and Differences Among Datasets

The eligible cases in the training set (data from hospitals 1 to 5) and the holdout test dataset (data from hospitals 1 to 5) exhibited significant differences in lesion subtype and American Joint Committee on Cancer (AJCC) stage when compared to the external test dataset (data from hospital 6; *P* < .001). However, no significant differences were observed in lesion localization, age, or Breslow thickness. The median (range) age at diagnosis was 58 (18-95) years for the training set, 57 (18-93) years for the holdout test dataset, and 61 (18-95) years for the external test dataset; the median (range) Breslow thickness was 0.70 (0.10-34.00) mm, 0.70 (0.20-14.40) mm, and 0.80 (0.30-20.00) mm, respectively. Thus, the training and holdout test datasets were considered to be differently distributed than the external one. Patient characteristics of the study sample are summarized in Table 2.

**Table 2.** Patient Characteristics of the Study Sample

Characteristic	Patients, No. (%)					
	Training set (hospitals 1-5)		Holdout test dataset (hospitals 1-5)		External test dataset (hospital 6)	
	IM (n = 209)	Nevus (n = 377)	IM (n = 37)	Nevus (n = 66)	IM (n = 142)	Nevus (n = 194)
Age at diagnosis, y						
<35	5 (2.4)	75 (19.9)	1 (2.7)	16 (24.2)	4 (2.8)	51 (26.3)
35-54	45 (21.5)	129 (34.2)	8 (21.6)	19 (28.8)	19 (13.4)	67 (34.5)
55-74	84 (40.2)	124 (32.9)	17 (45.9)	22 (33.3)	58 (40.8)	48 (24.7)
>74	74 (35.4)	49 (13.0)	11 (29.7)	9 (13.6)	61 (43.0)	28 (14.4)
Unknown	1 (0.5)	0	0	0	0	0
Lesion localization						
Palms/soles	1 (0.5)	6 (1.6)	1 (2.7)	3 (4.5)	4 (2.8)	5 (2.6)
Face/scalp/neck	43 (20.6)	17 (4.5)	8 (21.6)	4 (6.1)	24 (16.9)	26 (13.4)
Upper extremities	37 (17.7)	38 (10.1)	5 (13.5)	9 (13.6)	18 (12.7)	13 (6.7)
Lower extremities	45 (21.5)	78 (20.7)	8 (21.6)	13 (19.7)	29 (20.4)	34 (17.5)
Back	54 (25.8)	134 (35.5)	8 (21.6)	18 (27.3)	43 (30.3)	59 (30.4)
Abdomen	13 (6.2)	48 (12.7)	3 (8.1)	9 (13.6)	9 (6.3)	29 (14.9)
Chest	12 (5.7)	37 (9.8)	2 (5.4)	8 (12.1)	10 (7.0)	16 (8.2)
Buttock	2 (1.0)	10 (2.7)	1 (2.7)	2 (3.0)	1 (0.7)	5 (2.6)
Genitalia	1 (0.5)	5 (1.3)	1 (2.7)	0	1 (0.7)	3 (1.5)
Unknown	1 (0.5)	4 (1.1)	0	0	3 (2.1)	4 (2.1)
Lesion subtype						
Superficial spreading melanoma	142 (67.9)	NA	24 (64.9)	NA	35 (24.6)	NA
Nodular melanoma	25 (12.0)	NA	4 (10.8)	NA	20 (14.1)	NA
Lentigo maligna melanoma	29 (13.9)	NA	5 (13.5)	NA	9 (6.3)	NA
Acral lentiginous melanoma	8 (3.8)	NA	2 (5.4)	NA	6 (4.2)	NA
Desmoplastic melanoma	0	NA	0	NA	2 (1.4)	NA
Spitzoid melanoma	1 (0.5)	NA	1 (2.7)	NA	0	NA
Other types of IM/combined forms of IM/subtype unknown	4 (1.9)	NA	1 (2.7)	NA	70 (49.3)	NA
Spitz nevus and variants	NA	6 (1.6)	NA	0	NA	4 (2.1)
Dysplastic nevus/Clark nevus	NA	155 (41.1)	NA	30 (45.5)	NA	110 (56.7)
Acral nevus	NA	7 (1.9)	NA	4 (6.1)	NA	12 (6.2)
Recurrent nevus	NA	1 (0.3)	NA	0	NA	1 (0.5)
Blue nevus	NA	21 (5.6)	NA	3 (4.5)	NA	6 (3.1)
Other types of nevi/combined forms of nevi/subtype unknown	NA	187 (49.6)	NA	29 (43.9)	NA	61 (31.4)
AJCC stage^a^						
IA	87 (41.6)	NA	13 (35.1)	NA	70 (49.3)	NA
IB	23 (11.0)	NA	8 (21.6)	NA	30 (21.1)	NA
IIA	13 (6.2)	NA	3 (8.1)	NA	6 (4.2)	NA
IIB	7 (3.3)	NA	0	NA	14 (9.9)	NA
IIC	7 (3.3)	NA	3 (8.1)	NA	7 (4.9)	NA
IIIA	4 (1.9)	NA	0	NA	3 (2.1)	NA
IIIB	4 (1.9)	NA	0	NA	5 (3.5)	NA
IIIC	12 (5.7)	NA	1 (2.7)	NA	6 (4.2)	NA
IV	2 (1.0)	NA	1 (2.7)	NA	1 (0.7)	NA
Unknown	50 (23.9)	NA	8 (21.6)	NA	0	NA
Breslow thickness, mm^b^						
≤1.00 (T1)	126 (60.3)	NA	23 (62.1)	NA	89 (62.7)	NA
1.01-2.00 (T2)	25 (12.0)	NA	6 (16.2)	NA	16 (11.3)	NA
2.01-4.00 (T3)	27 (12.9)	NA	1 (2.7)	NA	19 (13.4)	NA
>4.00 (T4)	23 (11.0)	NA	6 (16.2)	NA	17 (12.0)	NA
Unknown	8 (3.8)	NA	1 (2.7)	NA	1 (0.7)	NA

Abbreviations: AJCC, American Joint Committee on Cancer; IM, invasive melanoma; NA, not applicable.

^a^ AJCC staging constitutes the criterion standard for histopathological reporting of IM.

^b^ Breslow thickness describes the extent of anatomic spread and serves as an important prognostic factor for IM.

### Comparison of FL With Other Approaches

To compare the performance of FL, a total of 586 lesions (209 IMs, 377 nevi) derived from 5 hospitals were used to train 3 distinct models (eFigure 1 in Supplement 1): first, the federated approach, where a model was built through decentralized training of individual models that were merged at regular intervals^36^; second, the centralized approach (Hfull), where a model was built using all available data on a centralized server^37^; and third, the ensemble approach, where a model was built for each participating hospital, and the results of all models were aggregated into one final prediction.^38^ A randomly sampled holdout test dataset from the same hospitals already involved in model training, consisting of 103 lesions (37 IMs, 66 nevi), and an external test dataset from another hospital not involved in model training, consisting of 336 lesions (142 IMs, 194 nevi), were used to evaluate the performances of the approaches.

### Performance of FL on Holdout Test Dataset

**Figure 2.**
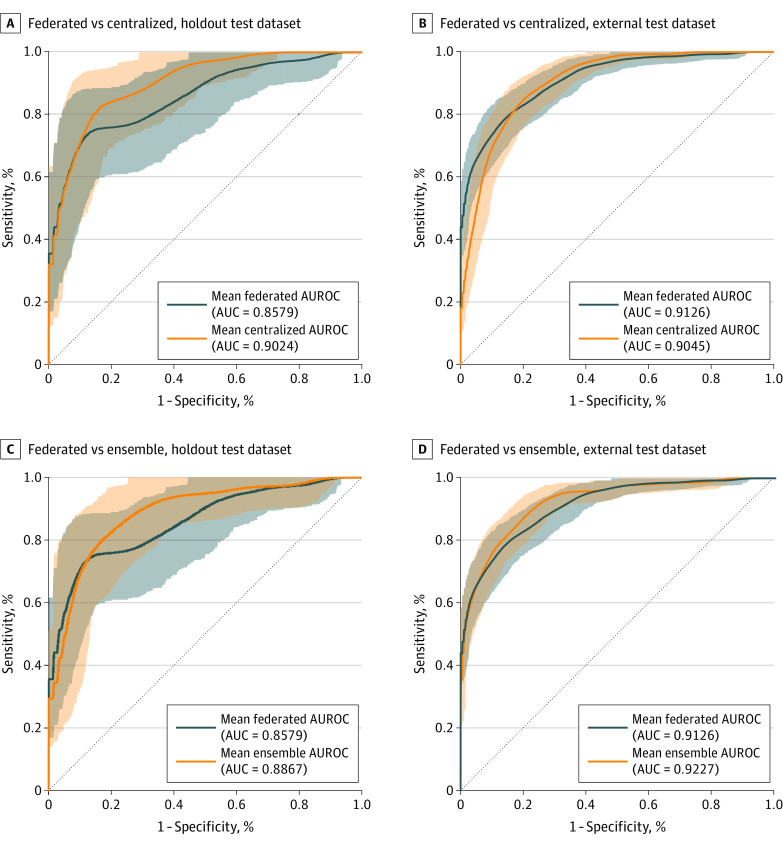
Mean Area Under the Receiver Operating Characteristic Curve (AUROC) of the 3 Investigated Approaches Mean AUROCs on the holdout and external test dataset after 1000 iterations of bootstrapping, including the corresponding 95% CIs (shaded areas), are illustrated for the federated learning (FL) and the centralized approach (model Hfull) (A and B) and for the FL and the ensemble approach (C and D). AUC indicates area under the curve.

On the holdout test dataset, FL performed the worst (Table 3), with a mean AUROC of 0.8579 (95% CI, 0.7693-0.9299; Figure 2), followed by the ensemble approach with a mean AUROC of 0.8867 (95% CI, 0.8103-0.9481). The centralized approach (model Hfull) performed best, with a mean AUROC of 0.9024 (95% CI, 0.8379-0.9565). The results indicate that on the holdout test dataset, the classical centralized model performed significantly better than the federated and ensemble approaches in terms of AUROC (pairwise Wilcoxon signed-rank, *P* < .001). For a detailed overview of the confusion matrices on the holdout test dataset, see eFigure 2 in Supplement 1.

**Table 3.** Performance Metrics of the Different Classification Approaches on the Holdout and External Test Datasets

Model	AUROC (95% CI)	Balanced accuracy, % (95% CI)	Sensitivity, % (95% CI)	Specificity, % (95% CI)
**Performance metrics of the different classification approaches**				
Holdout				
FL	0.8579 (0.7693-0.9299)	76.76 (67.70-84.89)	59.54 (42.86-75.00)	93.99 (87.84-98.55)
Ensemble	0.8867 (0.8103-0.9481)	81.46 (73.10-88.94)	84.02 (70.59-94.59)	78.89 (68.57-88.06)
Centralized	0.9024 (0.8379-0.9565)	85.23 (77.30-92.31)	83.91 (70.97-94.59)	86.55 (77.46-93.94)
External				
FL	0.9126 (0.8810-0.9412)	81.73 (77.36-85.77)	80.92 (74.21-86.90)	82.54 (77.07-87.92)
Ensemble	0.9227 (0.8941-0.9479)	76.47 (72.69-80.48)	95.79 (92.19-98.65)	57.16 (50.51-63.96)
Centralized	0.9045 (0.8701-0.9331)	80.56 (76.71-84.38)	93.66 (89.21-97.22)	67.46 (60.87-74.05)
**Performance metrics of the original federated approach and all 5 retrained leave-1-hospital-out approaches**				
Holdout				
FL	0.8579 (0.7693-0.9299)	76.76 (67.70-84.89)	59.54 (42.86-75.00)	93.99 (87.84-98.55)
H1	0.9139 (0.8508-0.9648)	79.30 (70.90-87.40)	67.59 (52.63-82.50)	91.02 (83.33-97.06)
H2	0.8874 (0.8041-0.9529)	82.76 (74.68-90.05)	72.91 (57.89-86.67)	92.61 (86.15-98.41)
H3	0.8675 (0.7879-0.9337)	74.15 (65.63-82.90)	54.23 (37.50-70.97)	94.06 (87.67-98.59)
H4	0.8851 (0.8099-0.9511)	81.55 (73.26-89.44)	81.19 (68.29-93.55)	81.91 (72.06-90.77)
H5	0.8710 (0.7961-0.9401)	84.10 (75.96-91.18)	89.24 (78.38-97.50)	78.95 (68.75-88.06)
External				
FL	0.9126 (0.8810-0.9412)	81.73 (77.36-85.77)	80.92 (74.21-86.90)	82.54 (77.07-87.92)
H1	0.8868 (0.8517-0.9207)	76.90 (72.60-80.99)	89.49 (84.09-94.24)	64.31 (57.43-70.77)
H2	0.8941 (0.8585-0.9252)	79.69 (75.59-83.84)	89.43 (84.29-93.92)	69.95 (63.37-76.22)
H3	0.8831 (0.8465-0.9172)	78.82 (74.30-82.76)	88.66 (82.99-93.48)	68.99 (62.43-75.13)
H4	0.8670 (0.8281-0.9020)	76.29 (71.84-80.39)	86.61 (81.21-91.88)	65.97 (59.28-72.77)
H5	0.8296 (0.7837-0.8698)	72.39 (67.77-76.60)	88.78 (83.45-93.63)	55.99 (49.46-62.78)

Abbreviations: AUROC, area under the receiver operating characteristic curve; FL, federated learning.

### Performance of FL on External Test Dataset

On the external test dataset, a different ranking was observed (Table 3). The centralized approach (model Hfull) performed the worst, achieving a mean AUROC of 0.9045 (95% CI, 0.8701-0.9331), while FL demonstrated a mean AUROC of 0.9126 (95% CI, 0.8810-0.9412; Figure 2). The ensemble approach performed the best on the external test dataset, with a mean AUROC of 0.9227 (95% CI, 0.8941-0.9479). Altogether, on the external test dataset, the FL approach yielded significantly better results than the centralized model in terms of AUROC (pairwise Wilcoxon signed-rank, *P* < .001). Notably, both the FL and centralized models performed significantly worse than the ensemble approach (pairwise Wilcoxon signed-rank, *P* < .001). For a detailed overview of the confusion matrices on the external test dataset, see eFigure 3 in Supplement 1.

### Comparison of FL With a More Realistic Centralized Approach

Furthermore, the classical centralized approach was subjected to retraining using several smaller datasets (models H1, H2, H3, H4, and H5) for comparison with the original federated approach, which was trained with all available training data. This comparison was conducted to investigate whether FL would achieve at least comparable results to centralized approaches when it had access to more data (ranging from 71 to 236 more cases). Thereby, we explored the feasibility of potential future clinical FL application scenarios where hospitals might be more willing to participate in the development and refinement of a classifier when no patient data need to be transferred to an external institution.

After retraining, the centralized approach maintained its superiority on the holdout test dataset in terms of AUROC regardless of which hospital was omitted for classifier training (models H1, H2, H3, H4, and H5; pairwise Wilcoxon signed-rank, *P* < .001; supporting data in Table 3). However, on the external test dataset, the model developed with the FL approach held its performance advantage over all 5 centralized models developed using smaller datasets (pairwise Wilcoxon signed-rank, *P* < .001; supporting data in Table 3). These results suggest that a surplus of training data does not necessarily result in superior classification performance for FL.

## Discussion

In this study, we aimed to develop and externally validate a decentralized trained FL model for melanoma-nevus classification using histopathological WSIs. Additionally, we directly compared FL with classical centralized and ensemble learning, which are commonly applied for melanoma classification tasks. In this context, FL achieved a mean AUROC of 0.8579 (95% CI, 0.7693-0.9299) on the holdout test dataset and 0.9126 (95% CI, 0.8810-0.9412) on the external test dataset, thus representing a reliable alternative.

The utilized datasets encompassed a comprehensive representation of IM cases encountered in day-to-day clinical care due to the prospective and consecutive data collection from multiple centers. By avoiding selection bias that may have arisen in previous melanoma classification studies that applied FL but collected data retrospectively,^22,23^ we minimized the risk of overestimating or underestimating the performance of the compared classifiers. A strength of our study is the long-tailed distribution of localizations and IM subtypes (including rare subtypes, such as spitzoid melanomas), and all possible AJCC stages and Breslow thickness categories.^39^ Training the model on such a heterogeneous dataset that captures the complexity of clinical IM data enables the model to effectively recognize lesions of different types, severity levels, and depths and allows the model to learn spatial patterns and specific characteristics associated with diverse body regions. This enhances its overall generalizability, ultimately leading to robust performance.

The data from hospital 6 served as an out-of-distribution test dataset, consisting of unseen data from an institution that was not part of the model training process. Notably, there were significant differences in the AJCC stages and lesion subtypes compared to the training and holdout datasets (Table 2). The data from hospital 6 included lesions from a slightly older patient group, specifically, more patients with IM older than 74 years. On the other hand, the holdout test dataset (ie, unshown data derived from hospitals 1 to 5) tended to contain slightly more lesions from the Breslow thickness categories T2 (1.01-2.00 mm) and T4 (>4.00 mm; Table 2). These differences may also be evident in the corresponding WSIs and could have influenced the performance of the evaluated approaches.

Overall, the classical centralized model (Hfull) significantly outperformed FL on the holdout test dataset (ie, tested on unshown data from hospitals involved in model training) in terms of AUROC (0.9024 vs 0.8579), while FL performed significantly better (0.9126 vs 0.9045) on the external test dataset (ie, on data from a hospital not involved in model training). The findings demonstrate that FL techniques may not be as well suited to solve in-distribution classification problems (ie, same distribution as the training data), as indicated by the inferior performance on the holdout test dataset. On the other hand, they show that FL may provide additional advantages in terms of out-of-distribution generalizability, as indicated by the enhanced performance on the external test datasets (similar to observations in Warnat-Herresthal et al^20^ and Dayan et al^25^). The observed superior performance on the external test set could be due to the FL model not fully converging during training, possibly introducing a slight regularization effect. This phenomenon of nonconvergence is frequently encountered in FL due to the challenging task of training on data from different distributions.^40^

While the observed differences between FL and the centralized approach may not be large in absolute terms, they are consistent over 1000 iterations of bootstrapping (ie, paired data comparisons), thereby demonstrating a sustained outperformance of the centralized approach. Despite the comparatively lower statistical power of the Wilcoxon signed-rank test, this marginal yet persistent performance improvement is clinically highly relevant, as any melanoma misclassification can lead to fatal outcomes.

Despite these positive findings, the ensemble approach continued to outperform FL and the classical centralized approach in terms of AUROC (0.9227 vs 0.9126 and 0.9045, respectively). Nevertheless, an ensemble approach poses extensive challenges for the explainability of the results, since understanding multiple sets of model weights is more difficult than dealing with 1 set in the FL approach. This is particularly relevant given the legislative requirement that medical devices must be explainable to a certain extent,^41^ as well as its substantial influence on patients’ and physicians’ acceptance.^42^

### Limitations

This study has limitations. Although the WSIs were digitized using the same slide scanner (Leica Aperio AT2 DX), heterogeneity was ensured by different staining and cutting protocols of the participating hospitals. While the labels for this study were established based on the criterion standard of care (ie, histopathological verification), caution should be exercised in interpreting the results, as previous studies observed a discordance between pathologists of up to 25% in classifying melanoma.^9,10^ Future studies may involve the integration of independent pathologist panels or epigenetic analyses (eg, methylation analyses) to further reduce interrater variability.

## Conclusions

The results of this diagnostic study demonstrate that FL can achieve a comparable performance to that of classical centralized or ensemble approaches, making it a reliable alternative for the classification of IMs and nevi. Additionally, FL empowers institutions to contribute to the development of AI models, even with relatively small datasets or strict data protection rules, thereby fostering collaboration across institutions and countries. Moreover, FL may have the potential to be further extended to other image classification tasks in digital cancer histopathology and beyond. Future research could build on this work by assessing its effectiveness with different types of medical images (eg, dermoscopic or hyperspectral images), evaluating its feasibility for diagnosing various types of cancer, and investigating its effectiveness using technically different (eg, attention-based methods) AI models. In our ongoing research, we are exploring the scalability of FL for refined diagnostic tasks by incorporating in situ tumors as a clinically highly relevant but separate classification class.

## Notes

Supplement 1. **eTable 1.** Dataset characteristics at the patch level. **eFigure 1.** Workflow of the three implemented approaches. **eFigure 2.** Confusion matrices of the three approaches on the holdout test dataset. **eFigure 3.** Confusion matrices of the three approaches on the external test dataset. **eTable 2.** The STARD 2015 list.

Supplement 2.
Data Sharing Statement
