# Supplementary material for: Federated Learning for Decentralized Artificial Intelligence in Melanoma Diagnostics
Source: JAMA Dermatol. 2024 Feb 7;160(3):303–11. doi: 10.1001/jamadermatol.2023.5550 (PMC10851139; doi:10.1001/jamadermatol.2023.5550)
Supplement: Supplement 1. — eTable 1. Dataset characteristics at the patch level. eFigure 1. Workflow of the three implemented approaches. eFigure 2. Confusion matrices of the three approaches on the holdout test dataset. eFigure 3. Confusion matrices of the three approaches on the external test dataset. eTable 2. The STARD 2015 list. [file jamadermatol-e235550-s001.pdf]

Supplemental Online Content

Haggenmüller S, Schmitt M, Krieghoff-Henning E, et al. Federated learning for decentralized artificial intelligence in melanoma diagnostics. *JAMA Dermatol.* Published online February 7, 2024. doi:10.1001/jamadermatol.2023.5550

eTable 1. Dataset characteristics at the patch level.

eFigure 1. Workflow of the three implemented approaches.

eFigure 2. Confusion matrices of the three approaches on the holdout test dataset.

eFigure 3. Confusion matrices of the three approaches on the external test dataset.

eTable 2. The STARD 2015 list.

This supplemental material has been provided by the authors to give readers additional information about their work.

© 2024 Haggenmüller S et al. *JAMA Dermatology.*

eTable 1. Dataset characteristics at the patch level.

**Hospital**	**Overall (#patches)**	**Melanoma (#patches)**	**Nevi (#patches)**
Hospital 1	80706	30180	50526
Hospital 2	32486	22910	9576
Hospital 3	51919	35693	16226
Hospital 4	85474	32226	53248
Hospital 5	80655	33748	46907
Hospital 6	217057	141384	75673
**Total**	**548297**	**296141**	**252156**

© 2024 Haggenmüller S et al. *JAMA Dermatology.*

eFigure 1. Workflow of the three implemented approaches.

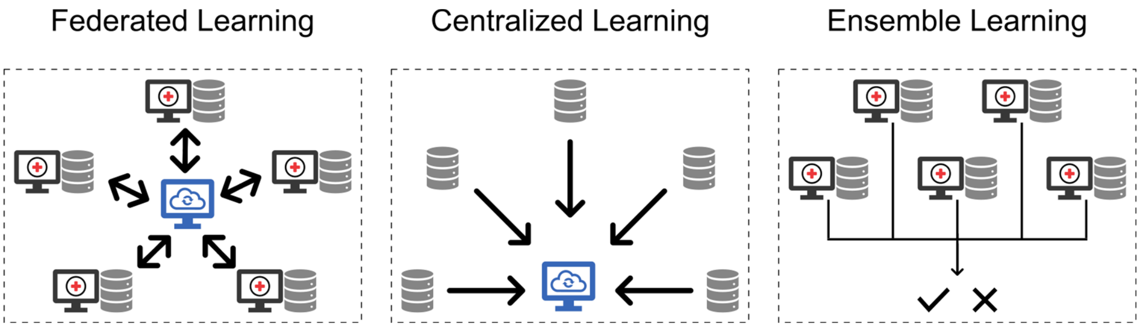

On the left side, the federated approach is depicted, where every hospital (represented by the red +) has its own data (gray database) and computing power (monitors), but communication and aggregation are executed by a third party (blue monitor) that serves as a central coordinator. In the middle, the centralized approach is represented. In this case, the hospitals transfer their data to a third party, which uses it to train a centralized model. On the right side, the ensemble approach is depicted, where each hospital uses their own data and computing power to train a separate model. The decisions over all models are averaged to obtain a final prediction for a given image.

eFigure 2. Confusion matrices of the three approaches on the holdout test dataset.

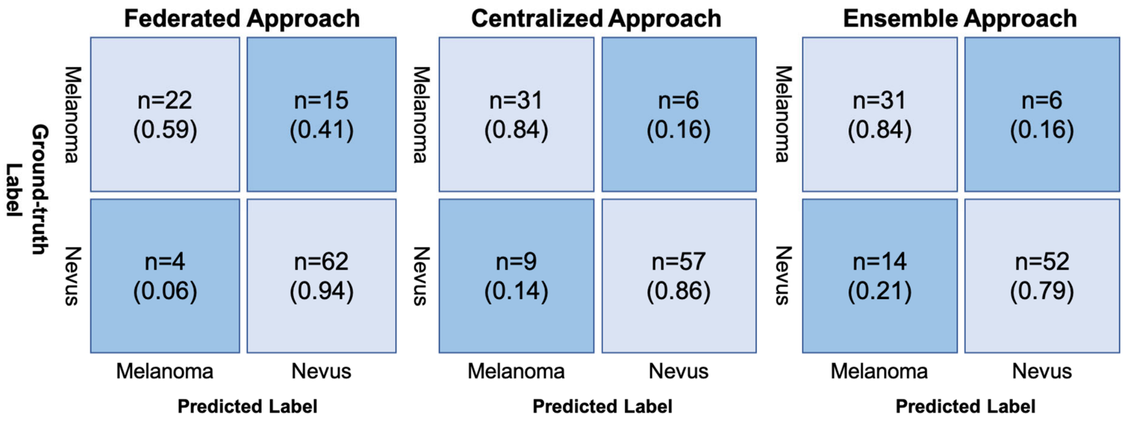

Distribution of correct and incorrect predictions on the holdout test dataset for the federated approach, the centralized approach H_{ful} and the ensemble approach. The ground truth was determined by at least one reference dermatopathologist at the corresponding hospital as part of routine clinical practice.

eFigure 3. Confusion matrices of the three approaches on the external test dataset.

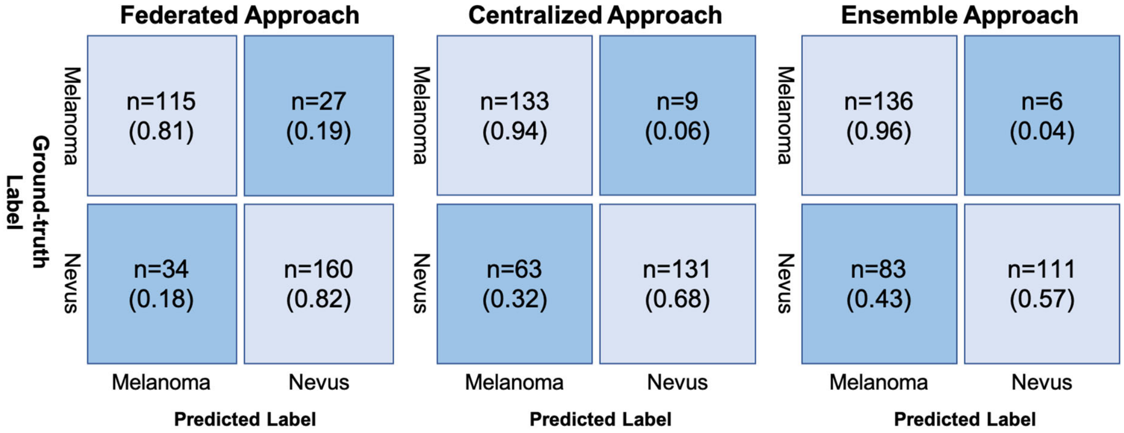

Distribution of correct and incorrect predictions on the external test dataset for the federated approach, the centralized approach H_{ful} and the ensemble approach. The ground truth was determined by at least one reference dermatopathologist at the corresponding hospital as part of routine clinical practice.

© 2024 Haggenmüller S et al. *JAMA Dermatology.*

eTable 2. The STARD 2015 list.

Topic	No	Item	Section in Manuscript
Title or abstract			
	1	Identification as a study of diagnostic accuracy using at least one measure of accuracy (such as sensitivity, specificity, predictive values, or AUC)	Abstract
Abstract			
	2	Structured summary of study design, methods, results, and conclusions	Abstract
Introduction			
	3	Scientific and clinical background, including the intended use and clinical role of the index test	Abstract,<br>Introduction
	4	Study objectives and hypotheses	Abstract,<br>Introduction
Methods			
Study Design	5	Whether data collection was planned before the index test and reference standard were performed (prospective study) or after (retrospective study)	Patient Cohorts and Slide Acquisition
Participants	6	Eligibility criteria	Patient Cohorts and Slide Acquisition
	7	On what basis potentially eligible participants were identified (such as symptoms, results from previous tests, inclusion in registry)	Patient Cohorts and Slide Acquisition
	8	Where and when potentially eligible participants were identified (setting, location, and dates)	Patient Cohorts and Slide Acquisition
	9	Whether participants formed a consecutive, random, or convenience series	Patient Cohorts and Slide Acquisition
Test methods	10a	Index test, in sufficient detail to allow replication	WSI Preprocessing,<br>Model Development,<br>Statistical Analysis
	10b	Reference standard, in sufficient detail to allow replication	WSI Preprocessing,<br>Model Development,<br>Statistical Analysis

© 2024 Haggenmüller S et al. *JAMA Dermatology.*

	11	Rationale for choosing the reference standard (if alternatives exist)	Introduction
	12a	Definition of and rationale for test positivity cut-offs or result categories of the index test, distinguishing pre-specified from exploratory	not applicable
	12b	Definition of and rationale for test positivity cut-offs or result categories of the reference standard, distinguishing pre-specified from exploratory	not applicable
	13a	Whether clinical information and reference standard results were available to the performers or readers of the index test	not applicable
	13b	Whether clinical information and index test results were available to the assessors of the reference standard	not applicable
Analysis	14	Methods for estimating or comparing measures of diagnostic accuracy	Statistical Analysis
	15	How indeterminate index test or reference standard results were handled	not applicable
	16	How missing data on the index test and reference standard were handled	Patient Cohorts and Slide Acquisition, Number of Eligible Slides and Patients
	17	Any analyses of variability in diagnostic accuracy, distinguishing pre-specified from exploratory	Statistical Analysis
	18	Intended sample size and how it was determined	Patient Cohorts and Slide Acquisition, Number of Eligible Slides and Patients
Results			
Participants	19	Flow of participants, using a diagram	Number of Eligible Slides and Patients
	20	Baseline demographic and clinical characteristics of participants	Patient Characteristics and Differences Among Datasets
	21a	Distribution of severity of disease in those with the target condition	Patient Characteristics and Differences Among Datasets
	21b	Distribution of alternative diagnoses in those without the target condition	Patient Characteristics and Differences Among Datasets
	22	Time interval and any clinical interventions between index test and reference standard	not applicable

© 2024 Haggenmüller S et al. *JAMA Dermatology.*

Test results	23	Cross tabulation of the index test results (or their distribution) by the results of the reference standard	Comparison of FL With Other Approaches
	24	Estimates of diagnostic accuracy and their precision (such as 95% confidence intervals)	Comparison of FL With Other Approaches
	25	Any adverse events from performing the index test or the reference standard	not applicable
Discussion			
	26	Study limitations, including sources of potential bias, statistical uncertainty, and generalisability	Discussion
	27	Implications for practice, including the intended use and clinical role of index text	Discussion
Other information			
	28	Registration number and name of registry	not applicable
	29	Where the full study protocol can be accessed	not applicable
	30	Sources of funding and other support; role of funders	Role of the Funder/Sponsor

© 2024 Haggenmüller S et al. *JAMA Dermatology.*
