# Supplementary material for: Federated Learning for Decentralized Artificial Intelligence in Melanoma Diagnostics
Source: JAMA Dermatol. 2024 Feb 7;160(3):303–11. doi: 10.1001/jamadermatol.2023.5550 (PMC10851139; doi:10.1001/jamadermatol.2023.5550)
Supplement: Supplement 2. — Data Sharing Statement [file jamadermatol-e235550-s002.pdf]

Data Sharing Statement

Haggenmüller. Federated Learning for Decentralized Artificial Intelligence in Melanoma Diagnostics. *JAMA Dermatol*. Published February 07, 2024.
doi:10.1001/jamadermatol.2023.5550

Data
Data available: No

Additional Information
Explanation for why data not available: The data that support the findings of this study will be part of the SCP2 research database. The data will be made available for the purpose of (skin) cancer research upon request and approval by the SCP2 consortium in the near future.
